# Medicines and Healthcare products Regulatory Agency’s “Consultation on proposals for legislative changes for clinical trials”: a response from the Trials Methodology Research Partnership Adaptive Designs Working Group, with a focus on data sharing

**DOI:** 10.1186/s13063-023-07576-7

**Published:** 2023-10-05

**Authors:** Martin Law, Dominique-Laurent Couturier, Babak Choodari-Oskooei, Phillip Crout, Carrol Gamble, Peter Jacko, Philip Pallmann, Mark Pilling, David S. Robertson, Michael Robling, Matthew R. Sydes, Sofía S. Villar, James Wason, Graham Wheeler, S. Faye Williamson, Christina Yap, Thomas Jaki

**Affiliations:** 1 Medical Research Council Biostatistics Unit, School of Clinical Medicine, University of Cambridge, Cambridge, UK; 2 Royal Papworth Hospital NHS Foundation Trust, Cambridge, UK; 3 Cancer Research UK Cambridge Institute, University of Cambridge, Cambridge, UK; 4 University College London, London, UK; 5 Liverpool Clinical Trials Centre, University of Liverpool, Liverpool, UK; 6 Lancaster University Management School, Lancaster University, Lancaster, UK; 7 Berry Consultants, Abingdon, UK; 8 Centre for Trials Research, Cardiff University, Cardiff, UK; 9 Department of Public Health and Primary Care, School of Clinical Medicine, University of Cambridge, Cambridge, UK; 10 British Heart Foundation Data Science Centre, Health Data Research UK, London, UK; 11 Biostatistics Research Group, Population Health Sciences Institute, Newcastle University, Newcastle upon Tyne, UK; 12 Imperial Clinical Trials Unit, Imperial College London, London, W12 7RH UK; 13 Clinical Trials and Statistics Unit, The Institute of Cancer Research, London, UK; 14 Faculty for Informatics and Data Science, University of Regensburg, Regensburg, Germany

**Keywords:** Consultation, Data sharing, Legislation

## Abstract

In the UK, the Medicines and Healthcare products Regulatory Agency consulted on proposals “to improve and strengthen the UK clinical trials legislation to help us make the UK the best place to research and develop safe and innovative medicines”. The purpose of the consultation was to help finalise the proposals and contribute to the drafting of secondary legislation. We discussed these proposals as members of the Trials Methodology Research Partnership Adaptive Designs Working Group, which is jointly funded by the Medical Research Council and the National Institute for Health and Care Research. Two topics arose frequently in the discussion: the emphasis on legislation, and the absence of questions on data sharing. It is our opinion that the proposals rely heavily on legislation to change practice. However, clinical trials are heterogeneous, and as a result some trials will struggle to comply with all of the proposed legislation. Furthermore, adaptive design clinical trials are even more heterogeneous than their non-adaptive counterparts, and face more challenges. Consequently, it is possible that increased legislation could have a greater negative impact on adaptive designs than non-adaptive designs. Overall, we are sceptical that the introduction of legislation will achieve the desired outcomes, with some exceptions. Meanwhile the topic of data sharing — making anonymised individual-level clinical trial data available to other investigators for further use — is entirely absent from the proposals and the consultation in general. However, as an aspect of the wider concept of open science and reproducible research, data sharing is an increasingly important aspect of clinical trials. The benefits of data sharing include faster innovation, improved surveillance of drug safety and effectiveness and decreasing participant exposure to unnecessary risk. There are already a number of UK-focused documents that discuss and encourage data sharing, for example, the Concordat on Open Research Data and the Medical Research Council’s Data Sharing Policy. We strongly suggest that data sharing should be the norm rather than the exception, and hope that the forthcoming proposals on clinical trials invite discussion on this important topic.

## Background

In the UK, the Medicines and Healthcare products Regulatory Agency (MHRA) consulted on proposals “to improve and strengthen the UK clinical trials legislation to help us make the UK the best place to research and develop safe and innovative medicines” [1]. The consultation took place from 17 January until 14 March 2022.

The proposals were discussed by the authors in their roles as members of the Medical Research Council (MRC) National Institute for Health and Care Research (NIHR) Trials Methodology Research Partnership Adaptive Designs Working Group (ADWG) [2]. While the ADWG did not respond directly to the consultation as an organisation, constituent members responded to the consultation individually, and we present this commentary as the consensus of the authors. We invited part of the Health Informatics Working Group, specifically, members of the data sharing subgroup, to comment on our document and thus they have been added as co-authors. The discussion of the proposals and their consequences was considerable and wide-ranging. We will not describe the group’s views on all 43 questions included in the consultation as the proposals have not yet been finalised. However, two topics were a repeated source of discussion: the role of legislation, and data sharing.

### Legislation

Firstly, the proposals place a considerable reliance on increased legislation (over alternatives such as funding support or educational work). While we believe legislation plays a part in driving good practice, we consider it most effective when it is tightly defined with clear goals in mind. For example, while we agree it is best practice to involve patients and the public in the different aspects of the trial, a proposal that simply “requires patient and public involvement” (Question 1) is likely to be ineffective at best and harmful at worst, with poor research practices being glossed over as “compliance”. In general, when legislation has to be interpreted in practice, one consequence can be tokenism. Furthermore, legislation can drive risk-averse practice rather than best practice.

### Data sharing

Secondly, the proposals do not address data sharing, a topic which the authors consider fundamental in clinical trials. This is the focus of the remainder of this short communication: the importance of data sharing in the landscape of modern clinical trials and its current absence from the proposals.

We define “data sharing” as making pseudonymised or linked individual-level or aggregate-level clinical trial data available to other investigators for further use.

A table listing benefits and beneficiaries of data sharing in the context of clinical trials is given by Mello et al. [3]. Benefits include faster innovation, improved surveillance of drug safety and effectiveness and decreasing participant exposure to unnecessary risk. Beneficiaries of the findings of data re-use projects include the scientific community, research participants and trial sponsors, as well as those who collect the data first-hand, such as research nurses and practitioners in the NHS. There are further benefits of data sharing specific to clinical trials [4–6]. These benefits include obtaining additional findings beyond the original trial outcomes, decreased duplication of work (leading to decreased expenses) and improved assumptions and sample sizes due to increased availability of historical data.

#### Data sharing in relation to adaptive designs

One point at which data sharing can positively affect the area of adaptive design clinical trials is at the earliest stage, before the trial begins: an adaptive design method may require data shared from historical trials, for example to form an informative prior distribution, in a level of detail that is uncommon [7]. Adaptive clinical trials may use a Bayesian framework, for example the continual reassessment method for early phase trials [8] or adaptive randomisation approaches for later phase trials [9, 10]. Bayesian methods use prior distributions, which can be chosen based on data obtained through data sharing. If adaptive clinical trial designs can be improved in theory but not in practice due to a lack of data sharing then this has negative consequences for clinical trials as a whole, as the potential advantages of adaptive designs (reduced sample size, faster decisions and so on) are lost.

## A wider view: open science policies and principles in the UK

Data sharing is just one aspect of the wider concept of open science and reproducible research. In the UK, there already exists a growing culture around open science or reproducible research, at least in the context of publicly-funded research, beginning with publishing papers as open access: UK Research and Innovation (UKRI) Open Access Policy (Version 1.4) states that all papers published featuring publicly-funded authors must be made immediately available as open access [11]. Similarly, all monographs, book chapters and edited collections must be made open access within 12 months of publication (from 1 January 2024). The Concordat on Open Research Data, published in 2016, describes open research data as “the next step in achieving the UK’s open science ambitions” [12]. Worldwide, some organisations have already proposed timelines for sharing data from published clinical trials [13].

The Concordat on Open Research Data as a whole consists of a set of ten principles detailing best practice for working with research data, with a particular focus on data sharing: not just why and how data sharing should be done, but also the associated difficulties [12].

The UKRI Open Access Policy already requires research articles to include a Data Access Statement, “even where there are no data associated with the article or the data are inaccessible”. The Open Access Policy states that data from publicly funded research (Annex 1) should be openly available, minimising restrictions [11], though this is not a requirement. This is based on the principles of the above Concordat on Open Research Data.

The MRC, part of UKRI, has a published Data Sharing Policy, consisting of twelve principles [14]. Similar to the UKRI Open Access Policy, the first principle states that data from MRC-funded research should be made available “with as few restrictions as possible”, though again this is not required. This policy was first published in 2005 and underwent minor changes in 2011, with the content unchanged since.

The National Institute for Health and Care Research (NIHR) has published a position on data sharing, initially in 2019 and updated in 2021 [15]. This position, for NIHR-funded studies, includes a requirement for data sharing statements regarding how to access the corresponding data, and requirements for data management and access plans are currently being introduced. The NIHR also has a database of open data [16].

### Data sharing opportunities and risks

Beyond publicly-funded research, the value of data sharing is recognised in the wider clinical trials community. This was shown by the creation of Clinical Study Data Request (CSDR), a consortium of pharmaceutical companies including GSK, Novartis and Bayer and research groups outside of industry, including some UK-based funders such as the MRC and Cancer Research UK [17]. CSDR is a platform for sharing of patient-level data from studies. We note that requests for data via this route have been fewer than expected, and data is not always accessed once access is granted [18]. A newer data sharing resource is Vivli, an independent, non-profit organisation with a data sharing platform, whose members again include a mixture of pharmaceutical companies and research groups [19]. For both CSDR and Vivli, requests may be denied by the company that developed the data. Addressing data sharing more generally, the FAIR Guiding Principles describe the various ways in which data should be “FAIR”, that is, Findable, Accessible, Interoperable and Reusable. In particular, the principles include making data retrievable by machines as well as people [20].

Hopkins et al. conducted a survey on data sharing among UK Clinical Research Collaboration (UKCRC)-registered, i.e. publicly funded Clinical Trial Units (CTUs), and found a mix of responses. While 16 of the 23 (70%) responding CTUs (out of a total of 45 surveyed) either had or were developing a data sharing policy, nine out of 21 (43%) responding CTUs gave specific reasons as to why their CTU could not adopt a standard data sharing policy [21]. Hopkins et al. conclude that “Adoption of a standard procedure, or at least some common principles across the CTUs, would greatly facilitate data sharing.” As an example of data sharing undertaken by an individual CTU, the MRC CTU at University College London has described its own independent approach as moderated access [22].

Prevalence of data sharing can be examined not only by CTU, but also by type of trial. A recent review of COVID-19 trials by Vanderbeek et al., 31/58 (53%) of platform trials planned to share individual participant data (IPD) [23]. Vanderbeek et al. compare this proportion to an even less favourable study by Danchev et al., where 334/487 (69%) of published trials committed to some degree of data sharing [24]. Of the 89 trials that committed to sharing IPD in repositories, only 17/89 (19%) had done so.

Data sharing comes with risks. For example: participant privacy; invalid or inappropriate secondary analyses as a result of accidental or intentional misuse of data, including as a consequence of data sharing “publication” bias (though the risk of publication bias itself is reduced if data are made available as a matter of routine, independently of the strength of results); disincentivising the development of new studies containing original data; lack of credit given to original investigators in subsequent analyses; increased strain on resources. These particular risks, and approaches to mitigating them, are discussed by Lo et al. [4].

## Conclusions

Strong data sharing policies can influence data availability: in an observational study of medRxiv preprints and their published counterparts, the proportion of manuscripts with openly shared data increased from 33.3% (at preprint) to 61.4% (at publication) for papers published in journals that mandate open data. The corresponding change for papers published in journals that do not mandate open data was 20.2 to 22.3% [25]. This demonstrates that mechanisms to increase data sharing, outwith legislation, do exist.

The increase in open access publications combined with existing data sharing opportunities, such as the CSDR, suggest that there is a willingness to embrace open data in the UK, within both publicly-funded research and pharmaceutical companies.

We do not prescribe a detailed proposal for guidelines and legislation on data sharing. However, we strongly suggest that the principle of data sharing should be the norm rather than the exception, strongly encouraged by both funders and journals and considered alongside all other aspects of clinical trials consulted on. Furthermore, this data sharing should take place within a reasonable time frame of publication of a clinical trial and be encouraged in upcoming proposals. The Concordat on Open Research Data provides a valuable, nuanced approach to best practice in data sharing, and we hope that the forthcoming proposals on clinical trials are in keeping with this approach.

## Data Availability

Data sharing is not applicable to this article as no datasets were generated or analysed during the current study.

## Abbreviations

ADWG: Adaptive Designs Working Group
CSDR: Clinical Study Data Request
CTU: Clinical Trials Unit
IPD: Individual Participant Data
MRC: Medical Research Council
MHRA: Medicines and Healthcare Products Regulatory Agency
NIHR: National Institute of Health Research
UKCRC: UK Clinical Research Collaboration
UKRI: UK Research and Innovation
