# Correction: Medicines and Healthcare products Regulatory Agency’s “Consultation on proposals for legislative changes for clinical trials”: a response from the Trials Methodology Research Partnership Adaptive Designs Working Group, with a focus on data sharing

**DOI:** 10.1186/s13063-023-07763-6

**Published:** 2023-11-21

**Authors:** Martin Law, Dominique-Laurent Couturier, Babak Choodari-Oskooei, Phillip Crout, Carrol Gamble, Peter Jacko, Philip Pallmann, Mark Pilling, David S. Robertson, Michael Robling, Matthew R. Sydes, Sofía S. Villar, James Wason, Graham Wheeler, S. Faye Williamson, Christina Yap, Thomas Jaki

**Affiliations:** 1 Medical Research Council Biostatistics Unit, School of Clinical Medicine, University of Cambridge, Cambridge, UK; 2 Royal Papworth Hospital NHS Foundation Trust, Cambridge, UK; 3 Cancer Research UK Cambridge Institute, University of Cambridge, Cambridge, UK; 4 University College London, London, UK; 5 Liverpool Clinical Trials Centre, University of Liverpool, Liverpool, UK; 6 Lancaster University Management School, Lancaster University, Lancaster, UK; 7 Berry Consultants, Abingdon, UK; 8 Centre for Trials Research, Cardif University, Cardif, UK; 9 Department of Public Health and Primary Care, School of Clinical Medicine, University of Cambridge, Cambridge, UK; 10 British Heart Foundation Data Science Centre, Health Data Research UK, London, UK; 11 Bio- Statistics Research Group, Population Health Sciences Institute, Newcastle Uni-Versity, Newcastle Upon Tyne, UK; 12 Imperial Clinical Trials Unit, Imperial College London, London, W12 7RH UK; 13 Clinical Trials and Statistics Unit, The Institute of Cancer Research, London, UK; 14 Faculty for Informatics and Data Science, University of Regensburg, Regensburg, Germany

**Correction: BMC Trials 24, 640 (2023)**

**https://doi.org/10.1186/s13063-023-07576-7**

Following publication of the original article [1], we have been informed that affiliation for Graham Wheeler was incorrectly presented. The correct affiliation at the time of manuscript submission is Imperial Clinical Trials Unit, Imperial College London.

Originally published affiliation: “Present address: Statistics & Data Science Innovation Hub, GSK, Brentford, UK”.

The original article has been corrected.
